# Adolescent’s subjective perceptions of chronic disease and related psychosocial factors: highlights from an outpatient context study

**DOI:** 10.1186/s12887-016-0748-x

**Published:** 2016-12-12

**Authors:** Teresa Santos, Margarida Gaspar de Matos, Adilson Marques, Celeste Simões, Isabel Leal, Maria do Céu Machado

**Affiliations:** 1 FMH, Faculdade de Motricidade Humana (Projecto Aventura Social-Social Adventure Team), Universidade de Lisboa, Estrada da Costa, 1495-688 Cruz Quebrada, Portugal; 2 ISAMB, Instituto de Saúde Ambiental, Faculdade de Medicina, Universidade de Lisboa, Lisbon, Portugal; 3 William James Center of Research, ISPA-Instituto Universitário, Ciências Psicológicas, Sociais e da Vida, Rua Jardim do Tabaco, n°34, 1149-041 Lisbon, Portugal; 4 CIPER, Centro Interdisciplinar de Estudo da Performance Humana, Faculdade de Motricidade Humana, Universidade de Lisboa, Lisbon, Portugal; 5 Departamento de Pediatria do Hospital de Santa Maria, CAML, Centro Académico de Medicina de Lisboa, FM, Faculdade de Medicina/Universidade de Lisboa, Av. Professor Egas Moniz, 1649-028 Lisbon, Portugal

**Keywords:** Adolescents, Chronic health conditions, Health-related quality of life, Psychosocial factors, Psychosomatic health, Resilience, Self-regulation, Social support

## Abstract

**Background:**

Adolescents with chronic disease (CD) can be more vulnerable to adverse psychosocial outcomes. This study aims: 1) to identify differences in psychosocial variables (health-related quality of life, psychosomatic complaints, resilience, self-regulation and social support) among adolescents who feel that CD affects or does not affect school/peers connectedness (measured by self-reported participation in school and social activities); and 2) to assess the extent to which psychosocial variables are associated with connectedness in school and peer domains.

**Methods:**

A cross-sectional study was conducted in 135 adolescents with CD (51.9% boys), average age of 14 ± 1.5 years old (*SD* = 1.5). Socio-demographic, clinical, and psychosocial variables were assessed, using a self-reported questionnaire, which included the Chronic Conditions Short Questionnaire, KIDSCREEN-10 Index, Symptoms Check-List, Healthy Kids Resilience Assessment Module Scale, Adolescent Self-Regulatory Inventory, and Satisfaction with Social Support Scale. Descriptive statistics, GLM-Univariate ANCOVA and Logistic Regression were performed using the IBM Statistical Package for Social Sciences (SPSS), version 22.0. The significance level was set at *p* < 0.05.

**Results:**

Thirteen to eighteen percent of the adolescents felt that CD affected participation at school (PSCH) and participation in leisure time with friends (PLTF). These adolescents presented lower results for all psychosocial study variables, when compared with adolescents who did not feel affected in both areas of participation. From the studied psychosocial variables, the most important ones associated with PSCH (after controlling for age, gender, diagnosis, and education level of father/mother) were self-regulation and psychosomatic health. Concerning the PLTF, social support was the sole variable explaining such association.

**Conclusions:**

The present study pointed out the association between psychosocial variables; and living with a CD and school/peers connectedness. The need to focus on the assessment of the effects of a CD on adolescents’ lives and contexts is suggested, as well as on the identification of vulnerable adolescents. Such identification could help to facilitate the maximization of social participation of adolescents with CD, and to plan interventions centered on providing support and opportunities for a healthy youth development. For that purpose, a complex and multifactorial approach that includes clinicians, schools, family, and peers may be proposed.

## Background

Adolescence is a critical developmental stage for a positive course of future health and well-being, especially in the context of living with a chronic disease (CD), which is often characterized by great variability in its definition, assessment, prevalence and impact on the child or adolescent [1]. Apart from this variability, it is well recognized that a CD can represent a major psychosocial burden, contribute to the risk of psychosocial stress [2] and unhealthy psychosocial development [3–5], as well as poor Quality of Life (QoL)/Health-related Quality of Life (HRQoL) [6–9], health and well-being [10].

Additionally, living with a CD can have consequences beyond the individual level, specifically in the academic context [11, 12], placing adolescents at higher risk for poor educational, vocational, and social outcomes [13], and leading to isolation from the peer group [14]. The participation in educational/social activities with peers and the connections with other people/institutions are crucial as age increases. Particularly in adolescence, since it assumes a major importance in the socialization process [15], representing a powerful positive protective factor and a key component for developing healthy youth [16, 17]. Peer relationships and support from close friends can also play a special significant role when a CD exists, constituting a great help to cope with the illness and with inherent psychosocial and lifestyles changes [18]. However, such participation can be weakened due to various consequences of having a CD in adolescence [19].

In spite of these findings showing an increased vulnerability in adolescents with CD, other studies have identified a significantly lower risk of impairment in QoL/HRQoL and in psychosocial functioning [17, 20, 21], highlighting the need to better understand such controversial results, through adolescents’ self-report. This is particularly relevant, because the process of adaptation to a chronic disease is heterogeneous, variable and depends on specific individual/contextual factors [22]. Moreover, situations of cumulative risk (when additional problems occur beyond the type of disease/emerging limitations), may have a strong impact and constitute a threat to the adolescent’s well-being [23, 24].

The literature also indicated that a dynamic interdependence exists between the adolescent and his environment including individual, socioeconomic and demographic factors [25]. In addition, as a child advances in age, physical and biomedical factors diminish their importance as determinants of self-perceived QoL, and psychosocial factors become relevant [26]. Thus, psychosocial variables assume an important role, more than the presence *per se* of physical dimensions of the health condition [1, 12, 27], and to address psychosocial dimensions is crucial for the holistic care of these children and adolescents [28–32]. Literature concerning the assessment of such dimensions, including research comparing children’s and parents’ QoL (Quality of Life) across several health conditions, and children with different health conditions [33, 34] has been identified. However, studies have mostly focused on proxy-reports [2, 35] and have not analyzed the influence of age-different groups (childhood and adolescence) on the outcomes, nor considered a consistent approach to age group specificities [2, 33, 36, 37].

Thus, to our knowledge, the effects of living with a chronic condition in the specific period of adolescence, focusing on self-perceptions and on connectedness and psychosocial factors, has not been extensively evaluated. Further research is needed because this is an important and relevant area of research for educators and clinicians, both in primary care and specialties.

Facing this scenario, the present study will rely on self-perceptions of adolescents and will focus on school and peers connectedness (participation at school, PSCH; and participation in leisure time with friends, PLTF), because both aspects have been shown to be associated with positive youth health outcomes. This choice is in agreement with previous studies pointing out the need to address limitations in ordinary activities of chronically ill adolescents [38], the need to focus less on diagnostic categories (where more variability exists) and more on psychosocial dimensions and on the effects that the chronic disease might have on the social activities/socialization and emotional health [1]. In addition, considering that QoL/HRQoL and psychosocial dimensions are related to the individual’s perceptions on the impact of diseases, the literature recommends to focus on self-reported instruments for a better measurement [38, 39] and the WHO guidelines [40] also emphasize its use whenever possible.

More specifically, the current study aims are: 1) to identify differences in psychosocial variables (health-related quality of life, psychosomatic complaints, resilience, self-regulation and social support) among adolescents who feel that CD affects or does not affect PSCH and PLTF; and 2) to assess the extent to which psychosocial variables are associated with connectedness in school and peer domains. It is hypothesized that adolescents living with a CD, and feeling that CD has a higher impact on their social participation (PSCH and PLTF), are more vulnerable to psychosocial health outcomes.

## Methods

### Participants, design and procedure

The present cross-sectional study included 135 adolescents, attending the pediatric outpatient department of a public central hospital, with diagnosis of diabetes *mellitus*, allergic or neurological diseases. The choice of these chronic conditions was based on its highly prevalence in adolescence [30, 41–43] and considering the fact that these chronic conditions were among the most commonly treated and of easier access at the recruitment site. Additionally it is highlighted that the present study takes into consideration the non-categorical approach, not analyzing the effects of the different specific CDs, but rather focusing on the general effects of having a CD in this sample. This relies on the literature that pointed out that despite biological differences, the experience of living with a CD has similarities concerning psychosocial challenges and consequences for adolescents [44–47]. It is also in agreement with a “within-group” study perspective, relevant to identify factors that better predict social adjustment or disease management [18].

Prior to data collection, an ethical approval for this study was obtained from The Ethics Committee for Health and the institution’s ethical committees. Using a convenience sampling technique, adolescents were directly approached and selected by their health professionals (physicians and/or nurses) during the different medical expertise appointments. The following inclusion criteria were applied: 1) diagnosis of chronic disease, which was established by a physician and also ascertained in the questionnaire; 2) ages ranging from 12 to 16 years old; and 3) to have the cognitive skills necessary to fill out the questionnaire autonomously. Following the World Medical Association Declaration of Helsinki’s guidelines proposed in 2013, detailed information about the study aims and procedures was provided to all participants; those who met the inclusion criteria were invited to participate. The participation was voluntary and the agreement and informed consent was obtained, both from parents and adolescents. Data were collected (whenever possible in an individual medical office) using a self-reported questionnaire, either after or before the medical appointment, according to the most opportune moment for all. Research assistance was available to provide support whenever necessary. Adolescents completed the questionnaire themselves, in accordance with literature that has been gradually steering away from the practice of seeking opinion through proxy from parents or healthcare providers. This perspective considers that adolescents are good interpreters of their experiences on health, health-related needs and feelings [2, 48], as well as competent interpreters of their “world” [49], along with an increasing tendency to “give voice” to children/adolescents with chronic diseases [50, 51].

### Measures

All the following measures were obtained in a single self-reported questionnaire that took approximately 45 min to respond.

Socio-demographic and clinical variables were attained, namely age, gender, geographic region, nationality, and education level (adolescents and parents). To define a chronic condition, support from the assistant pediatrician was required, and the questionnaire additionally included the question “Do you have a long-term disability, illness or medical condition that has been diagnosed by a doctor? No/Yes”. In addition, the impact of a chronic health condition on the adolescents’ activities was assessed by the following questions: 1) “Does your long-term illness, disability or medical condition affect your attendance and participation at school? No/Yes” (PSCH) and, 2) “Does your long-term illness, disability or medical condition affects your attendance and participation in leisure activities with friends, classmates? No/Yes” (PLTF). These questions were previously used in an optional package of the international study Health Behaviour in School-aged Children (HBSC/WHO) [52, 53], and in the Portuguese survey HBSC/WHO [43], also constituting the Chronic Conditions Short Questionnaire (CCSQ) [48]. This instrument helps to understand the psychosocial impact of chronic illnesses, and shows considerable strengths over a single question, open-ended item. Co-existing problems related to the need to take medication, and/or missing school classes, are also reported as good indicators of severity. The adolescents were also asked some specific questions related to the disease: the time since diagnosis, the use of special equipment, and the use of medication related to the disease.

Psychosocial variables were also obtained including Health-related Quality of Life (KIDSCREEN-10 Index), Psychosomatic Health Complaints (Symptoms Check-List), Resilience (Scale Healthy Kids Resilience Assessment Module), Self-regulation (Scale Adolescent Self-Regulatory Inventory-ASRI), and Social Support (Scale of Satisfaction with Social Support-SSSS). Detailed information on interpretation of these measures is described in Table 1.

**Table 1:** Psychosocial variables

Name	Psychosocial measure	Abbreviation (in this study)	Short description
KIDSCREEN-10 Index [58]	Health-related quality of life – HRQoL	KIDS-10	• Short version of KIDSCREEN-52; • Used in the HBSC/WHO Study [43, 52, 53]; • 10 items, on a 5-point Likert-type scale; • Ranges from 0 to 100; • Lower values reflect feelings of unhappiness, dissatisfaction and inadequacy. Higher values reveal feelings of happiness, perception of adequacy and satisfaction with the adolescent’s life contexts. • α = .83
Symptoms Check List (SCL-HBSC) [59]	Psychosomatic health complaints (unidimensional latent trait).	SCL	• Used in the HBSC/WHO Study [43, 52, 53]; • 8 items focusing on subjective physical and psychological health complaints; • Each item answered on a 5-point Likert-type response scale; • Resultant values between 1 (worst health) and 5 (best health); • Ranges from 8 to 40. • α = .78
Healthy Kids Resilience Assessment Module [60]	Resilience (2 dimensions: external and internal resources).	RES	• The present study only considered the internal resources dimension; • 18 items answered on a 4-point scale; • Ranges from 18 to 72; • Higher scores indicate higher levels of competences, protection and resilience to adversity. • α = .0.72
Adolescent Self-Regulatory Inventory – ASRI [61]	Self-regulation (2 dimensions: Short term-SR-ST and Long term-SR-LT).	SR	• In this study the instrument was translated from the original English version into Portuguese language. It was then revised by a group of specialized experts within this field and a pre-test with a group of students was conducted in schools. • 36 items answered on a 5-point Likert scale; • Ranges from 36 to 180. • Higher values indicate better competences of self-regulation. • α = .0.79
Scale of Satisfaction with Social Support [62, 63]	Satisfaction with social support (2 dimensions: Satisfaction with Social Support-SSS; and Need for Activities connected to Social Support-NASS).	SSSS	• Translation and adaptation for children and adolescents of a Satisfaction with Social Support Scale for adults [63]; • 12 items answered on a 5-point scale; • Ranges from 18 to 72; • Higher scores indicate higher satisfaction with social support (SSS) or higher satisfaction for not feeling the need to have more social support activities (NASS). • α = .85

### Statistical analysis

Descriptive statistics were calculated for demographic, clinical, and psychosocial variables (means, standard deviation, and percentages) for the total group of adolescents. All data were tested for normality prior to any analyses using Shapiro-Wilk and Kolmogorov-Smirnov tests, as well as Levene's test for the homogeneity of the variance. A GLM-Univariate ANCOVA (analysis of covariance) was conducted to determine differences between adolescents who felt that CD affects or does not affect participation at school and participation in leisure time with friends on psychosocial variables, controlling for standard demographic variables such as age, gender, diagnosis of chronic condition, and education level acquired by both father and mother.

Later, a logistic regression was used to assess the extent to which psychosocial variables were associated with affecting and not affecting school and leisure time with friend’s participation. The group “not feeling affected” was used as the reference group. Adjusted and unadjusted odds ratio (OR) with 95% confidence intervals (CIs) were calculated and the significance level was set at *p* < 0.05. To avoid multicollinearity in the logistic regression analysis, variables were tested and none was omitted. All statistical analyses were completed using IBM Statistical Package for Social Sciences (SPSS), version 22.0. The significance level was set at *p* < 0.05.

## Results

The 135 included adolescents (51.9% boys; 48.1% girls) had a mean age of 14 years (*SD* = 1.5), and had three diagnosed chronic diseases: diabetes *mellitus* (*n* = 43), allergic diseases (*n* = 63), and neurologic diseases (*n* = 29). The majority of these adolescents lived in the Lisbon area (*n* = 114), attended the 7^th^-9^th^ grades (*n* = 72) and had a Portuguese nationality (*n* = 132). They also had 7.0 years of median time of diagnosis, generally did not use special equipment (*n* = 83), mostly took medication (*n* = 88) for their chronic disease and felt that living with a CD did not affect PSCH (*n* = 111), or PLTF (*n* = 117).

The socio-demographic and the clinical variables included in the study for the total group of adolescents are presented in Table 2.

**Table 2:** Socio-demographic and clinical characteristics for the total group of adolescents with chronic disease

	Total group
	*N* = 135
Age (years) (M ± SD)	14.0 ± 1.5
Diagnosis (%)	
Diabetes *mellitus*	31.9
Allergic Diseases	46.7
Neurological Diseases	21.5
Gender (%)	
Boy	51.9
Girl	48.1
Education Level - Father (%)	
Basic Level (1^st^-9^th^ Grades)	64.6
Secondary Level (10^th^-12^th^ Grades)	23.6
Superior (or more) Level (University, Post-Graduate)	11.8
Education Level - Mother (%)	
Basic Level (1^st^-9^th^ Grades)	53.8
Secondary Level (10^th^-12^th^ Grades)	29.5
Superior (or more) Level (University, Post-Graduate)	16.7
“Does your long-term illness, disability or medical condition affects your attendance and participation at school?” (%) - PSCH	
No	82.2
Yes	17.8
“Does your long-term illness, disability or medical condition affects your attendance and participation in leisure activities with friends, classmates?” (%) - PLTF	
No	86.7
Yes	13.3

The comparisons on all psychosocial variables, between affecting/not affecting PSCH and between affecting/not affecting PLTF, are shown in Table 3. The group of adolescents who feel that CD does not affect PSCH showed a higher health-related quality of life compared to the group that felt that CD affects PSCH (82.2 ± 10.1 *vs*. 68.0 ± 15.6; *F*(1,118) = 33.16, *p* < .001). Also, those adolescents present a better psychosomatic health (36.7 ± 3.8 *vs*. 30.3 ± 5.8; *F*(1,118) = 42.46, *p* < .001), higher resilience (59.0 ± 7.4 *vs*. 55.5 ± 9.1; *F*(1,118) = 6.07, *p* = .015), higher self-regulation competences (121.8 ± 14.4 *vs*. 110.9 ± 10.6; *F*(1,118) = 10.78, *p* = .001), and more social support (46.6 ± 7.6 *vs*. 38.2 ± 10.0); *F*(1,118) = 22.72, *p* < .001), when compared with the individuals who felt that CD affects PSCH. The group of adolescents who felt that CD does not affect PLTF reported a higher health-related quality of life when compared to the group that felt that CD affects participation (81.6 ± 10.6 *vs.* 67.3 ± 16.6; *F*(1,118) = 22.53, *p* < .001). In addition, those adolescents showed a better psychosomatic health (36.2 ± 4.2 *vs*. 31.6 ± 6.6; *F*(1,118) = 11.61, *p* = .001), higher resilience (59.0 ± 7.4 *vs*. 54.3 ± 9.1; *F*(1,118) = 4.22, *p* = .042), higher self-regulation competences (121.2 ± 14.3 *vs.* 111.8 ± 12.7; *F*(1,118) = 5.90, *p* = .017), and more social support (46.6 ± 7.5 *vs*. 35.3 ± 9.2; *F*(1,118) = 38.32, *p* < .001), when compared with the ones who feel that CD affects PLTF.

**Table 3:** Comparison of psychosocial study variables according to chronic disease affecting/not affecting participation at school (PSCH) and affecting/not affecting participation in leisure time with friends (PLTF)

	ADOLESCENTS WITH CD (M ± SD)						
	Total	Not affects PSCH	Affects PSCH	*p*	Not affects PLTF	Affects PLTF	*P*
KIDS-10	79.7 ± 12.5	82.2 ± 10.1	68.0 ± 15.6	<0.001***	81.6 ± 10.6	67.3 ± 16.6	<0.001***
SCL	35.6 ± 4.8	36.7 ± 3.8	30.3 ± 5.8	<0.001***	36.2 ± 4.2	31.6 ± 6.6	0.001**
RES	58.4 ± 7.8	59.0 ± 7.4	55.5 ± 9.1	0.015*	59.0 ± 7.4	54.3 ± 9.1	0.042*
SR	120.0 ± 14.4	121.8 ± 14.4	110.9 ± 10.6	0.001**	121.2 ± 14.3	111.8 ± 12.7	0.017*
SR-ST	41.8 ± 6.8	42.7 ± 6.7	37.6 ± 5.7	0.001**	42.4 ± 6.7	37.9 ± 5.9	0.011*
SR-LT	50.2 ± 7.7	51.1 ± 7.7	46.2 ± 6.1	0.013*	50.7 ± 7.7	46.8 ± 7.0	0.061
SSSS	45.1 ± 8.6	46.6 ± 7.6	38.2 ± 10.0	<0.001***	46.6 ± 7.5	35.3 ± 9.2	<0.001***
NASS	15.8 ± 4.7	16.5 ± 4.4	12.6 ± 4.4	0.001**	16.4 ± 4.3	12.0 ± 4.9	<0.001***
SSS	29.1 ± 5.4	30.0 ± 4.3	25.3 ± 7.6	<0.001***	30.03 ± 4.3	23.3 ± 7.7	<0.001***

Tested by GLM – Univariate ANCOVA

Analyses were adjusted for age, gender, diagnosis of chronic disease and educational level – father and mother

*CD* Chronic Disease, *PSCH* Participation at School, *PLTF* Participation in Leisure Time with Friends, *SCL* Symptoms Check List, *KIDS* KIDSCREEN, *RES* Resilience, *SR* Self-regulation, *SR-ST* Self-regulation short term, *SR-LT* Self-regulation long term, *SSSS* Social Support, *NASS* Need for Activities connected to social support, *SSS* Satisfaction with social support

***p < .001; **p < .01; *p < .05

Table 4 shows the results of the unadjusted and adjusted results of the logistic regression analysis across the different psychosocial variables and CD not affecting PSCH/PLTF, including the total group of adolescents.

**Table 4:** Logistic regression analyses with odds ratios (OR) and 95% confidence intervals (CI) of the psychosocial study variables and not feeling that chronic disease affects PSCH nor PLTF

	CD Does not affects PSCH		CD Does not affects PLTF	
	Unadjusted	Adjusted	Unadjusted	Adjusted
	OR (95% CI)	OR (95% CI)	OR (95% CI)	OR (95% CI)
KIDS-10	1.11 (1.06–1.17)***	1.04 (0.96–1.13)	1.10 (1.05–1.16)***	1.03 (0.93–1.14)
SCL	1.31 (1.16–1.47)***	1.32 (1.12–1.57)**	1.16 (1.05–1.28)**	1.02 (0.86–1.21)
RES	1.08 (1.01–1.15)*	0.91 (0.82–1.02)	1.07 (1.00–1.15)	0.94 (0.82–1.07)
SR	1.08 (1.03–1.13)**	1.08 (1.02–1.15)**	1.07 (1.02–1.12)**	1.05 (0.97–1.12)
SSSS	1.14 (1.07–1.22)***	1.06 (0.96–1.17)	1.26 (1.13–1.41)***	1.23 (1.08–1.40)**

Analysis were adjusted for age, gender, diagnosis of chronic condition and educational level – father and mother

*CD* Chronic Disease, *PSCH* Participation at School, *PLTF* Participation in Leisure Time with Friends, *KIDS* KIDSCREEN, *SCL* Symptoms Check List, *RES* Resilience, *SR* Self-regulation, *SSSS* Social Support

***p < .001; **p < .01; *p < .05

In the unadjusted analysis, higher health-related quality of life was associated with higher odds of belonging to the group of adolescents who did not feel affected in PSCH (OR 1.11; CI 95% 1.06–1.17, *p* < 0.001). Additionally, adolescents perceiving a better psychosomatic health (reporting less symptoms) (OR 1.31; CI 95% 1.16–1.47, *p* < 0.001), resilience (OR 1.08; CI 95% 1.01–1.15, *p* < 0.05), self-regulation (OR 1.08; CI 95% 1.03–1.13, *p* < 0.01), and social support (OR 1.14; CI 95% 1.07–1.22, *p* < 0.001) were associated with higher odds of belonging to the group of adolescents who did not feel affected in PSCH. The results of the adjusted regression analysis, when all variables were introduced into the model (and after adjusting for age, gender, diagnosis of chronic condition and education level of father and mother), showed that better psychosomatic health (OR 1.32; CI 95% 1.12–1.57, *p* < 0.01) and not feeling that the CD affects PSCH, maintained an association with higher odds of belonging to the group of adolescents who did not feel affected in PSCH, though it was slightly less significant. Self-regulation also maintained an association with higher odds (OR 1.08; CI 95% 1.02–1.15, *p* < 0.01). In turn, health-related quality of life, resilience, and social support were no longer significant for the group of adolescents after the adjustment.

The unadjusted results showed that higher levels for all of the psychosocial variables, except for resilience, were associated with higher odds of belonging to the group of adolescents who did not feel affected in PLTF. Higher health-related quality of life (OR 1.10; CI 95% 1.05–1.16, *p* < 0.001), psychosomatic health (reporting less symptoms) (OR 1.16; CI 95% 1.05–1.28, *p* < 0.01), self-regulation (OR 1.07; CI 95% 1.02–1.12, *p* < 0.01) and social support (OR 1.26; CI 95% 1.13–1.41, *p* < 0.001) showed an association with higher odds of belonging to the group of adolescents who did not feel affected in PLTF. The results of the adjusted regression analysis, when all variables were introduced into the model (and after adjusting for age, gender, diagnosis of chronic condition and education level of father and mother), showed that higher social support (OR 1.23; CI 95% 1.08–1.40, *p* < 0.01) and feeling that the CD does not affect PLTF maintained an association with higher odds. In turn, all the other psychosocial variables, namely health-related quality of life, symptoms check-list, resilience, and self-regulation were no longer significant for the group of adolescents after the adjustment.

## Discussion

The first aim of this study was to identify differences in psychosocial variables between adolescents who felt that CD affects or does not affect PSCH and PLTF. Focusing on the socio-demographic and clinical variables, it was found that the majority of the adolescents reported that living with a CD does not affect participation at school (PSCH); neither does it affect the participation in leisure time with friends (PLTF). These findings are consistent with previous research that showed that the majority of adolescents with chronic diseases are satisfied with their personal and social lives, while some found the disease challenging [1]. However, despite the results in the present study, a substantial number of adolescents (13-18%) expressed that CD affects PSCH and PLTF, and it can be suggested that these group may need more support in adapting to the effects of the disease, compared to their peers. Statistically significant differences were also found, for all psychosocial variables, between adolescents who felt that CD affects/does not affect PSCH. Higher health-related quality of life, better psychosomatic health, higher resilience, higher self-regulation competences, and more social support were observed in the group of adolescents who felt that CD did not affect PSCH, when compared to ones who felt that CD affects PSCH. The same significant differences were found for all psychosocial variables among adolescents who felt that CD affects/does not affect PLTF. These results support literature that pointed out that the effects of living with a CD in adolescence can be extended to other contexts (e.g., school, and peer relationships) beyond the individual level [11–13], and that the consequences of the disease may weaken the connections between adolescents, other people, and institutions, leading to higher risk of decreased participation in educational and social activities [14, 19].

Therefore, it may be suggested that for healthy psychosocial functioning, the impact of the illness assumes a crucial importance [1, 34]. Furthermore, in the present study lower scores on psychosocial variables were associated with the group of adolescents who felt that the CD affected PSCH and PLTF, and this supports literature indicating that these adolescents can be more vulnerable to higher risk for psychosocial outcomes [2–5, 10] and QoL/HRQoL [6–9]. It is also in line with the idea in the literature that in the presence of cumulative risks (e.g., having CD and feeling it affects PSCH/PLTF), the impact on the adolescent’s well-being can be stronger [23, 24].

The second aim of this study focused on assessing the psychosocial variables that were associated with CD affecting or not affecting both areas of participation (PSCH and PLTF). Concerning PSCH, the adolescents who reported a higher health-related quality of life, better psychosomatic health, higher resilience, higher self-regulation, and more social support were more likely to have a significant association with the group that felt that CD did not affect school participation. However, the results show that after controlling for age, gender, diagnosis of chronic condition, and education level of father/mother, self-regulation and psychosomatic health were the most important psychosocial variables to explain such association. The importance of self-regulation is found in previous research, suggesting that living and adapting to a CD involves adherence to multiple complex daily tasks, and that all these demands require a high level of self-regulation in order to improve health outcomes [54]. Therefore, the adolescents who reported higher self-regulation concerning their CD were more likely to feel that it does not affect PSCH. Self-regulation can be somehow also related to disease management strategies. This is an increasingly important area, defined in literature, and fundamental to reduce symptoms in most chronic diseases. It may be suggested proposed to focus these strategies not only on the medical domains, but also in connection with the psychosocial needs of young people with CD [55]. Therefore, the promotion of self-regulation skills could be a focus of intervention programs for adolescents who feel that CD has an impact on their PSCH [56]. Better psychosomatic health was also considered to be an important variable having an association with adolescents who felt that the CD does not affect PSCH. Such results can be explained taking into account the literature that indicated that to have psychosomatic complaints could represent an additional burden for adolescents already living with the difficulties of a CD [4]. To have these possible effects in mind is a key aspect that health professionals who deal with chronically ill adolescents would need to be aware of, in order to better target the treatment and improve the management of the CD. Still relying on the literature, an additional explanation can be related to the fact that adolescents with less severe medical conditions or treatments, and with better health, may express less concerns about the social impact and possible disruption of their friendships [18, 19], and also be at a lower risk of experiencing restrictions compared to other adolescents [25]. Therefore, the ones with more difficulties in various psychosocial variables may avoid social situations or activities, which may lead to poorer school attendance or lack of participation in peer-group interactions [11, 12, 14].

Regarding PLTF, all psychosocial variables except resilience (higher health-related quality of life, better psychosomatic health, higher self-regulation, and better social support) had a significant association with the group of adolescents who felt that the CD did not affect participation in leisure time with friends. However, the results show that, after controlling for age, gender, subgroup of chronic condition and education level of father/mother, social support was the sole and most important variable explaining this association. These results are in accordance with literature that suggests that social support plays a crucial role in the presence of a CD [15, 18], that connections with peers are key aspects for a healthy youth [16, 17], and that participation in social activities can improve QoL. Literature also points out that the inclusion of adolescents’ close friends and peer relations in the disease management (i.e., increase friend’s support and recruit them to facilitate health-promoting behaviors), and in school reentry programs, can be important actions that facilitate disease adaptation and prevent friendships from being disrupted, when youngsters miss school due to the illness. Previous research states that these issues could also be addressed by health care providers within the medical setting [18].

According to these results and in agreement with the literature, it is crucial to address relevant psychosocial areas [1, 12, 26–32] in the specific age group of adolescents [2, 33, 36, 37] and in their life contexts, because it may give a clear rationale for the implementation of effective interventions, in order to increase social skills, social support and to maximize successful participation [12, 25]. It may be also considerate to adopt an integral perspective focused on the holistic care of these adolescents [28–32]. Such suggestions are in line with the adolescent-friendly health service concept [57], which suggests the inclusion of physical, psychological, and social perspectives.

This study has some limitations. The sample used was not representative and plausible generalizations should take this into consideration. Self-reported data might introduce recall bias, and due to the heterogeneity of the group of adolescents (different diseases/limitations), some are likely to be underrepresented. The cross-sectional design of the study precludes inferences concerning causality, offering a weak basis to examine the direction of the effects. Longitudinal data would be needed. Nevertheless, this study has numerous strengths, such as providing information concerning psychosocial variables and school/peers connectedness, in an outpatient context with adolescents with CD. Other strengths include the use of self-reported information from the adolescent’s themselves and not through proxy-reports, and the use of well-validated measures for health-related quality, psychosomatic health, resilience, and social support assessment. It would be relevant to replicate these variables in a larger sample, and in specific populations, in forthcoming research.

## Conclusions

The present study highlighted the association between the effects of living with a chronic condition and school/peers connectedness, and the relationship between several key psychosocial factors. There are a few implications. Considering that adaptation responses can be quite varied, and well-being can go beyond mere medical aspects (diagnosis/severity), it might be worthwhile for clinicians to turn their attention to the assessment of the impact of a chronic condition on adolescents’ lives and contexts. This suggestion is valid because it has been underlined that there are a substantial number of adolescents who feel that the disease affects their PSCH and PLTF; they seem to be at a higher risk in their psychosocial well-being. Once these vulnerable adolescents are identified, interventions, which focus on providing psychosocial support and opportunities for healthy youth development, can be implemented. Ultimately, the importance of a complex and multifactorial approach that includes clinicians, schools, family, and peers is emphasized.

## Abbreviations

CCSQ: Chronic conditions short questionnaire
CD: Chronic disease
HBSC/WHO: Health Behaviour in School-aged Children/World Health Organization
HRQoL: Health-related quality of Life
KIDS: KIDSCREEN
NASS: Need for activities connected to social support
PLTF: Participation in leisure time with friends
PSCH: Participation at school
QoL: Quality of Life
RES: Resilience
SCL: Symptoms check list
SR: Self-regulation
SR-LT: Self-regulation long term
SR-ST: Self-regulation short term
SSS: Satisfaction with social support
SSSS: Social support.
